# Profile of blood cells and inflammatory mediators in periodic fever, aphthous stomatitis, pharyngitis and adenitis (PFAPA) syndrome

**DOI:** 10.1186/1471-2431-10-65

**Published:** 2010-09-06

**Authors:** Kelly L Brown, Per Wekell, Veronica Osla, Martina Sundqvist, Karin Sävman, Anders Fasth, Anna Karlsson, Stefan Berg

**Affiliations:** 1 Department of Rheumatology and Inflammation Research, Sahlgrenska Academy at the University of Gothenburg, Gothenburg, Sweden; 2 Department of Pediatrics, Sahlgrenska Academy at the University of Gothenburg, Gothenburg, Sweden; 3 Department of Pediatrics, NU-Hospital Organization, Uddevalla, Sweden; 4 The Queen Silvia Childrens Hospital, Gothenburg, Sweden

## Abstract

**Background:**

This study aimed to profile levels of blood cells and serum cytokines during afebrile and febrile phases of periodic fever, aphthous **s**tomatitis, pharyngitis and adenitis (PFAPA) syndrome to advance pathophysiological understanding of this pediatric disease.

**Methods:**

A cohort of patients with a median age of 4.9 years experiencing 'typical PFAPA' episodes participated in this study. Blood cells and serum cytokines were analyzed by CBC analysis and multiplex ELISA.

**Results:**

Oscillations in the concentration of blood cells during the afebrile and febrile phases of typical PFAPA syndrome were observed; novel findings include increased monocytes and decreased eosinophils during a febrile episode and increased thrombocytes in the afebrile interval. Relatively modest levels of pro-inflammatory cytokines were present in sera. IFNγ-induced cytokine IP10/CXCL10 was increased after the onset of fever while T cell-associated cytokines IL7 and IL17 were suppressed during afebrile and febrile periods.

**Conclusions:**

Identification of dysregulated blood cells and serum cytokines is an initial step towards the identification of biomarkers of PFAPA disease and/or players in disease pathogenesis. Future investigations are required to conclusively discern which mediators are associated specifically with PFAPA syndrome.

## Background

The *p*eriodic *f*ever, *a*phthous stomatitis, *p*haryngitis and cervical *a*denitis (PFAPA) syndrome was originally described by Marshall *et al *in 1987 and the acronym, PFAPA, was coined two years later together with the diagnostic criteria [1,2]. PFAPA syndrome is regarded as a non-hereditary disease of unknown etiology although the clinical observation is that, in a small proportion of cases, one of the parents or a more distant relative had similar symptoms in childhood [3]. The actual incidence of PFAPA syndrome in the pediatric population is not known but it is more common than the hereditary periodic fevers, except in populations with an ethnic origin in the Eastern Mediterranean basin where FMF is more common. Every pediatrician is likely to encounter at least one case of PFAPA during his or her career [4]. Autoinflammatory attacks in PFAPA syndrome occur within a 2-8 week interval with remarkable clockwork periodicity in approximately 50% of patients [3,5,6]. As in other periodic fever syndromes, there is a marked increase in CRP, SAA, ESR and leukocyte concentration [6-8], which are indicative of a prominent, acute inflammatory reaction. In PFAPA, these indicators return to normal levels, symptoms subside and patients resume daily activities between episodes [9]. Although the episodes are self-limiting in PFAPA without any known, increased risk of sequel or mortality, the recurrent episodes have a major impact on the daily life of the entire family. Treatment options aim to reduce febrile symptoms and typically include NSAIDs and paracetamol [5]. Steroid treatment will most often abort an episode within hours, yet tends to reduce the length of the symptom-free interval [3,10] Tonsillectomy has been correlated with the resolution of the disease in a majority of cases [11-14]. Irrespective of treatment options, an inevitable cessation of the steadfast periodicity of attacks will occur on average 4.5 years after the first attack [5]. The spontaneous remission of the syndrome is independent of antibiotic, anti-inflammatory, or immunosuppressive treatment [5,15]. Our experience however, is that a considerable proportion of patients continue to have episodes for several years after their "recovery". These episodes are often mild and not always reported.

At present, the diagnosis of PFAPA is made on the basis of clinical phenotype, detailed case history and an exclusion of other diseases, including, infections, immunodeficiencies, autoimmune diseases and monogenic periodic fevers. Five clinical criteria must be fulfilled in order to diagnose PFAPA syndrome ([5]; see also *Subjects and Methods*). Additional, discriminatory features that may aid in the differential diagnosis of PFAPA include the duration and remarkable clockwork periodicity of inflammatory attacks. Episodes typically last 4-5 days in PFAPA, which is similar to HIDS (3-6 days), longer than FMF (6 hours-3 days) and shorter than TRAPS (>7 days) [2,5,6,8,15,16]. The prompt resolution of an episode in response to corticosteroids is another typical feature of PFAPA that is often considered when reaching a diagnosis [5,10].

To further advance the pathophysiological understanding of the disease, experimental investigations at the cellular and molecular level are required. This study was designed with the intended purpose to profile blood cell and serum cytokine levels during both afebrile and febrile phases of PFAPA syndrome.

### Subjects and Methods

#### Participants

Ten children with diagnosed PFAPA were selected to participate in this study based on age, ethnicity and clinical presentation. Participants 1) were less than 7 yr. of age, 2) fulfilled the standard clinical criteria for PFAPA syndrome [5], namely i) consistent recurring fevers from an early (<5 years) age, ii) symptoms in the absence of upper respiratory tract infection with at least one of the following clinical signs: aphthous stomatitis, cervical lymphadenitis or pharyngitis, iii) exclusion of cyclic neutropenia, iv) asymptomatic clinical phenotype between febrile episodes, and v) normal growth and development, 3) had febrile episodes lasting 3-5 days followed by 4) an asymptomatic interval between attacks (on average, 3-5 weeks) and 5) lacked additional features that would suggest a hereditary periodic fever syndrome: skin rash, arthritis, severe abdominal pain, diarrhea, thoracic pain and splenomegaly, fever episodes longer than 7 days, a history of hearing loss or symptoms secondary to cold exposure [6,17]. Moreover, patients with an ethnic origin in the Eastern Mediterranean basin, the Netherlands, Belgium or France were excluded to further minimize the risk of including patients with hereditary FMF or MKD. To our knowledge MKD has never been genetically diagnosed in a population of Swedish ethnic origin. Children received clinical care at the Queen Silvia Children's Hospital (Gothenburg, Sweden) or Uddevalla County Hospital, (Uddevalla, Sweden). If there was any doubt that fever on the day of sampling was not caused by a PFAPA episode, a pediatrician in the field assessed the patient; otherwise patients were not routinely assessed at the time of sampling. The patients did not receive steroids or prophylactic treatment during febrile episodes or afebrile intervals either during the study or immediately preceding their participation in the study. Controls were recruited among children that were admitted to either hospital for minor surgery. Approval for the study was obtained from the Regional Ethical Review Board at the University of Gothenburg, Sweden. Informed consent from the parents of patients and controls was obtained in accordance with the Declaration of Helsinki.

#### Samples

15 ml of blood from patients and controls were collected in Vacutainer^® ^tubes containing heparin or EDTA or, for the isolation of sera, clot activators (Becton Dickinson). Blood was collected from patients after the commencement of fever (febrile sample) and a minimum of 7 days after the termination of fever (afebrile sample). With the exception of two patients, blood was collected from all febrile patients within 24 hours of fever onset. For the two outstanding patients, blood was collected from one patient (P04) 120 hours after fever began while the other patient (P03) developed fever 12 hours after the blood was collected. Sera were obtained by centrifugation of blood at 1200-1500 revolutions per minute (rpm) for 10 minutes (mins). For cytokine determinations (see below), sera were stored in sterile tubes at -80°C prior to analysis.

#### Complete blood cell count and acute phase reactant levels

A complete blood cell count (CBC) and white blood cell (WBC) differential was determined using an ADVIA Cell counter. Acute phase proteins (CRP and SAA) and procalcitonin were measured by ELISA (Clinical Immunology Laboratory, Sahlgrenska University Hospital, Gothenburg).

#### Isolation and culture of human peripheral blood mononuclear cells (PBMC)

PBMC from patients and controls were prepared as previously described [18]. In brief, whole blood was separated by centrifugation over Ficoll at 1000 rpm, 30 mins at 4°C. PBMC were isolated from the buffy layer, washed and suspended in cold PBS. PBMC were seeded at 1 × 10^6 ^cells/ml in 96-well V-bottom plates (2.5 × 10^5 ^cells/well; Brandtech Scientific Inc) and incubated 24 hours at 37°C, 5% CO_2 _in RPMI-1640 medium supplemented with 10% heat-inactivated FCS, 2 mM L-glutamine, 1 mM sodium pyruvate and 50 Units/ml PEST (PAA Laboratories). Plates were centrifuged at 1000 rpm, 5 mins to pellet cells after which supernatants were transferred to sterile 96-well plates and stored at -80°C until analysis.

#### Detection of inflammatory mediators

Inflammatory mediators were analyzed in a subset of serum samples and supernatants using a 25-Plex inflammatory cytokine kit (Biosource International Inc) [19] and Luminex 100™ StarStation software (Applied Cytometry Systems) as described [18]. To minimize variability in cytokine levels due to the time of sampling, three febrile samples acquired at a similar time after the onset of fever (~15 hr.) were selected for multiplex analysis. An IP10 cytokine bead array assay (BD Biosciences) was used according to manufacturer's protocols to confirm multiplex results on a larger sample set.

#### Statistical Analysis

Statistical evaluation of data was performed by non-parametric one-way ANOVA with post-hoc analysis (Tukey's). A *p*-value < 0.05 was considered statistically significant; * indicates *p *< 0.05, ** *p *< 0.01 and *** *p *< 0.001. Medians are indicated by horizontal lines in the figures. Mean fold change ± SEM are reported in the text.

## Results

### Participants have "typical PFAPA" syndrome

Children diagnosed with PFAPA syndrome according to the standard clinical criteria [5] were included in this study. They were assessed by a pediatrician experienced in the field and were described as having "typical PFAPA". This designation was based primarily on clinical grounds; patients had recurring fever for, on average, 4 days with a history of regular intervals between febrile episodes and symptoms consistent with the acronym for PFAPA. Moreover patients lacked symptoms of, and ethnic predisposition to, hereditary fevers (see Subjects and Methods for details). The median age of the patients was 4.9 years, ranging from 11 months to 6.8 years. Beyond this age, febrile flares often occur with less frequency and intensity and, in almost all cases, eventually cease altogether [5]. Controls were recruited from otherwise healthy children that were admitted to hospital for minor surgery. The median age of this group was 5.2 years, ranging from 19 months to 9.5 years. Characteristics of patients and controls are described in Table 1 (patients) and Additional File 1, Table S1.

**Table 1:** Clinical description of PFAPA patients

^a^ID	Gender (F/M)	Allergy (+/-)	^b^Length of episode (d)	^c^Interval between episodes (wk)	^d^PFAPA characteristics			Other signs or symptoms during episodes	^e^Age (yr;mo) at			^f^Febrile sample (h)
								
					A (+/-)	P (+/-)	CA (+/-)		onset	diagnosis	sampling (AF/F)	
P01	F	-	3-5	3-5	+	+	+	Occasional vomiting	0;2	0;11	0;11	
P02	M	-	4-5	3-4	+	+	+		0;8	1;5	1;8	
P03	F	-	3-4	4	+	+	+	Leg pain	2;3	3;6	3;8	-12
P04	M	+	3-4	3-4	+	+	+		4;5	5;1	5;2/5;2	120
P05	M	-	3-4	4-5	-	+	+		3;4	4;2	4;2/4;2	15-17
P06	F	-	5	4-6	+	+	+		1;9	5;4	6;9/6;10	13-15
P07	M	-	4	4	+	+	+	Sporadic abdominal pain	1;6	3;10	5;6/5;5	15-18
P08	F	-	3	4	+	+	-	Frequent abdominal pain	1;6	4;8	5;3/5;2	15
P09	M	-	4-5	2-3	+	+	+	Sporadic abdominal pain & arthralgia	1;7	4;7	4;7	15-17
P10	F	+	4	3-4	+	+	+	Sporadic vomiting & abdominal pain	2;1	4;11	6;0	12-17

^a^Each individual was given an identification number (ID) where the numerical digit is unique to each individual.

^b^Duration of episodes as reported by the parents in days (d)

^c^Interval between episodes (from first to first day in successive episodes) as reported by the parents in weeks (wk)

^d^Symptoms of PFAPA according to the acronyms, A; Aphthous stomatitis, P; Pharyngitis, CA; Cervical adenitis

^e^Age at onset as reported by the parents. Age at diagnosis was collected from medical records. Age at in the AF: Afebrile or F: Febrile period. Age reported in years (yr) and months (mo).

^f^The number of hours (h) that the patient had fever before samples were taken

### Acute phase proteins CRP and SAA are elevated

Serum concentrations of C-reactive protein (CRP) and serum amyloid A (SAA) were substantially elevated in patients with fever (Table 2 and Additional File 2, Figure S1) which is typical for the syndrome. Worthy of note are the elevated levels of SAA in some afebrile PFAPA samples, suggesting that SAA is an extremely sensitive inflammatory marker. It has been suggested that procalcitonin may be used to distinguish fever that arises from PFAPA from fever associated with a bacterial infection, the latter of which would correlate with procalcitonin concentrations above 0.2 μg/L (http://www.kliniskkemi.se and [20]). Accordingly, procalcitonin levels in sera from afebrile patients (AFP), febrile patients (FP) and controls were less than this reference concentration (Table 2 and Additional File 2, Figure S1). Even at these low concentrations there was a significant difference in procalcitonin levels between FP sera and AFP/control sera. Lastly, all participants were free from overt signs of infection at the time of sampling and serum levels of endotoxin were similar in AFP, FP and controls (data not shown), providing additional evidence that PFAPA syndrome was the cause of fever in our cohort.

**Table 2:** Acute phase proteins in serum

		^e^CRP	^f^SAA	^g^Procalcitonin
	^d^ID	mg/L	mg/L	μg/L
	C01	<5	<11	0.06
	C02	0.0	<11	0.05
	C03	0.3	<11	0.10
	C04	0.2	<11	0.06
	C05	0.1	<11	0.07
	C06	0.1	20	0.16
^a^control	C07	0.2	<11	<0.05
	C08	0.4	53	0.09
	C09	1.9	<11	0.12
	C10	1.0	14	0.06
	C11	0.4	<11	<0.02
	C12	3.5	28	0.06
	C13	0.6	<11	0.09
	C14	0.5	<11	0.06

	P01	<5	15	0.06
	P02	3.3	13	0.04
	P03	1.2	16	0.10
^b^afebrile	P04	**10.7**	26	0.14
(AF)	P05	1.0	<11	0.05
	P06	0.5	<11	0.05
	P07	3.3	59	0.03
	P08	0.5	12	<0.05

	P05	**>75**	**>600**	0.21
	P06	**>75**	**560**	0.12
	P07	**>75**	**560**	0.08
^c^febrile	P08	**44**	**>600**	0.10
(F)	P09	**67**	**>600**	0.14
	P10	**75**	**>600**	0.10
	P03	1.22	11	0.09
	P04	**>75**	**590**	0.41

Data shown as scatter plots in Additional File 2, Figure S1

^a-d^Concentration of acute phase proteins in sera from ^a^healthy children and PFAPA children in either an ^b^afebrile interval or ^c^within the first 20 hours of a febrile episode. Samples from FP03 and FP04 were drawn respectively ࿄ 12 hours before and ࿄120 hours after fever appeared. Numerical digits in the assigned ^d^identification number (ID) are unique to individuals. Values in bold are outside the range for healthy children http://www.kliniskkemi.se.

^e^CRP; C reactive protein. Upper limit of detection is 75 mg/L. Concentration in healthy children is <5 mg/L.

^f^SAA; serum amyloid A. Lower and upper limits of detection are 11 and 600 mg/L. Concentration in healthy children is <11 mg/L.

^g^In the absence of a bacterial infection, the concentration of procalcitonin is <0.2 μg/L.

### The abundance of thrombocytes, neutrophils, monocytes, lymphocytes and eosinophils undulate within a PFAPA cycle

**Figure 1:**
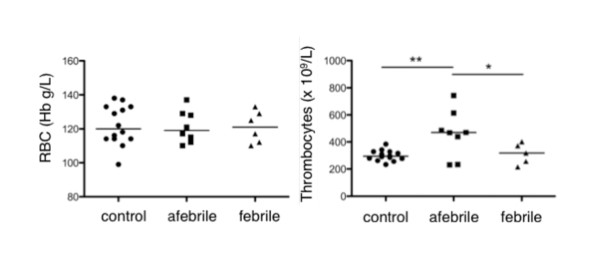
**Red blood cells and thrombocytes**. Red blood cells (RBC; measured as g/L of hemoglobin (Hb), left) and thrombocytes (x 10^9^/L, right) in blood from controls (n = 14), afebrile patients (n = 7-8) and patients with fever 12-18 hours (febrile, n = 6) were enumerated. Absolute cell numbers are reported in Additional File 3, Table S2.

**Figure 2:**
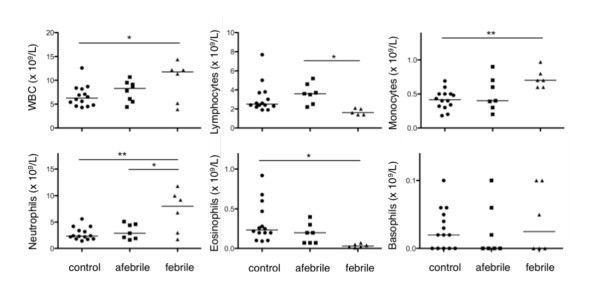
**Scatter plot of white blood cells**. The abundance (x 10^9^/L) of white blood cells (WBC; top left), lymphocytes (top centre), monocytes (top right), neutrophils (bottom left), eosinophils (bottom centre) and basophils (bottom right). Absolute cell numbers are reported in Additional File 3, Table S2.

The concentration of RBC, thrombocytes, WBC and constituent WBC subtypes (monocytes, lymphocytes, neutrophils, basophils and eosinophils) were evaluated in blood drawn from controls and patients. Data are presented in Figure 1, Figure 2 and Additional File 3, Table S2. The concentrations of both RBC and WBC in AFP samples were comparable to that in controls. The absolute numbers of thrombocytes in AFP blood however exceeded the upper limit of the normal range and were 1.5 ± 0.1 fold higher than thrombocytes in blood from FP and controls.

In FP blood, the absolute numbers of leukocytes was elevated (1.5 ± 0.2 fold) compared to the absolute numbers of leukocytes in control samples (Figure 2 and Additional File 3, Table S2). Thus, FP blood exhibited mild leukocytosis in contrast to controls but, by definition, clinical leukocytosis is not a defining characteristic of the febrile episodes. Two notable exceptions were febrile patients P05 and P06 that did not have elevated WBC at the time of sampling even though the febrile episode was consistent with previous febrile attacks and CRP and SAA levels were elevated. The increase in FP leukocytes corresponded with an increased absolute neutrophil count (ANC) and absolute monocyte count (AMC), which were elevated 2.6 ± 0.4 and 1.8 ± 0.1 fold, respectively, over concentrations in control blood. It is important to note that while the elevated ANC and AMC in FP blood were statistically distinguishable from controls, they nevertheless fell within normal ranges (Additional File 3, Table S2). While absolute lymphocyte counts (ALC) also tended to fall within the normal range, there was a substantial decrease in ALC in FP blood (65% less) compared to AFP samples. CBC analysis also revealed a major, unexpected difference in absolute eosinophil count (AEC) with less eosinophils in each of the FP samples (89% less) than in AFP samples.

### Classic pro-inflammatory cytokines TNFα and IL1β are not elevated in febrile sera

**Figure 3:**
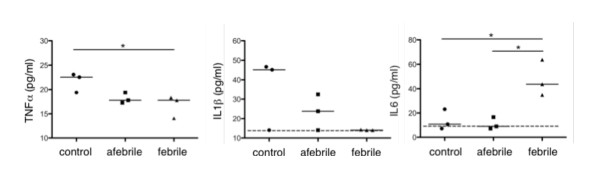
**Classic pro-inflammatory cytokines**. The concentrations (pg/ml) of prototypic pro-inflammatory cytokines TNFα (left), IL1β (centre) and IL6 (right) in sera from controls (n = 3), afebrile patients (n = 3) and febrile patients ~15 hours after the onset of fever (n = 3) were measured using a multiplex bead ELISA. The lower limits for detection of IL1β and IL6 are indicated by a dotted line. Measured quantities are reported in Additional File 4, Table S3.

A multiplex bead ELISA was used to determine the concentration of classic pro-inflammatory cytokines TNFα, IL1β and IL6 in FP sera that was drawn at approximately the same time after the onset of fever (~15 hours, n = 3). IL-6 was significantly increased in FP sera compared to control sera. The levels of TNFα and IL1β in all samples approached the lower limits of detection in the multiplex assay. Nevertheless, TNFα and IL1β concentrations were similar or slightly repressed in FP sera compared to control sera (Figure 3 and Additional File 4, Table S3). Given that the expression of IL-6 is often induced by TNFα or IL1β [21] and these cytokines were found in PFAPA sera 4 hours after the onset of fever [22], we anticipate that TNFα and IL1β peak early in the fever period then quickly approach homeostatic levels. A rapid oscillation of TNFα and IL1β *in vivo *occurs in response to infection [23,24].

### Th2- and Th17-associated cytokines are suppressed

**Figure 4:**
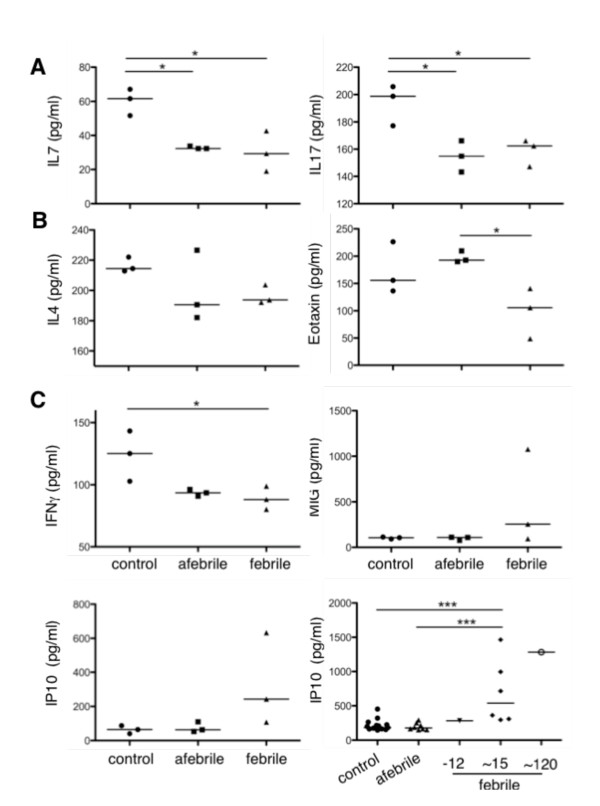
**T-helper-associated cytokines and chemokines**. The concentration (pg/ml) of (**A**) lymphocyte activator IL7 (left), Th17-associated cytokine IL17 (right) (**B**) Th2-associated cytokine IL4 and chemokine CCL11/Eotaxin (left and right respectively) and (**C**) Th1-associated cytokines and chemokines IFNγ (top left), MIG/CXCL9 (top right), and IP10/CXCL10 (bottom left) were measured in sera from controls (n = 3), afebrile patients (n = 3) and febrile patients ~15 hours after the onset of fever (n = 3) using a multiplex bead ELISA. Levels of IP10 were also assessed by a single-plex bead ELISA (**C**, bottom right) in controls (n = 13), afebrile patients (n = 8) and febrile patients 12 hours before, ~15 hours and ~120 hours after the onset of fever (-12 (n = 1), ~15 (n = 3), ~120 (n = 1)). Measured quantities are reported in Additional File 4, Table S3.

The presence of other pro-inflammatory cytokines was also investigated in sera by multiplex ELISA. The lymphocyte-specific cytokine, IL7, and CD4^+ ^T-helper cytokine, IL17, were suppressed in both AFP and FP sera compared to controls (Figure 4A and Additional File 4, Table S3). IL17 is produced primarily by a subset of CD4^+ ^T-helper-17 (Th17) cells. The activation of particular lymphocyte subsets, namely Th1, Th2 and Th17, is evident by the presence of particular cytokines that simultaneously drive one Th response while repressing others. The prototypic Th2 cytokine IL4 was present at comparable concentrations in AFP, FP and control sera. Concentrations of IL13, a Th2-associated cytokine, and CCL11/Eotaxin, a Th2-associated chemokine, were suppressed in sera from FP compared to controls (IL13) and AFP (IL13 and CCL11) sera (Figure 4B and Additional File 4, Table S3). The lower concentrations of these serum cytokines and IL17 suggested that neither a Th17- nor a Th2-type inflammatory response were active in FP sera.

### Th1-associated chemokine IP10 is elevated

The expression of the classic Th1-associated cytokines IFNγ and IL12 were not elevated in sera from AFP, FP and controls. The concentrations however of the IFNγ-induced chemokines CXCL9/MIG and CXCL10/IP10 were elevated, albeit to varying degrees, in sera from FP (Figure 4C and Additional File 4, Table S3). An independent bead-based ELISA specific for IP10 was used to validate the elevated concentration of IP10 in FP samples (AFP n = 8, FP n = 8, controls n = 13). The results revealed that IP10 was indeed elevated (*p *< 0.001) in sera taken ~15 hours and even as late as 120 hours after the onset of fever. Elevated levels of the IFNγ-induced chemokines CXCL9/MIG and CXCL10/IP10 in the absence of Th2- and Th17-associated cytokines indicated that components of a Th1-type inflammatory response coincide with fever in PFAPA syndrome.

### PBMC are not intrinsically activated in PFAPA

Since the AMC was elevated in FP blood and monocytes produce, among others, IL6, CXCL9/MIG and CXCL10/IP10, we questioned if cytokine production by isolated PBMC could reflect the cytokine profiles observed in sera. This approach has been used to prove that blood cells from individuals with HIDS, a monogenic periodic fever syndrome, and CGD, a condition in which sterile inflammatory disorders develop, are inherently active [25,26]. PBMC from AFP, FP and controls were cultured *ex vivo *for 24 hours following which the cell supernatants were evaluated by multiplex ELISA for the spontaneous secretion of the same pro-inflammatory cytokines that were evaluated in sera. No significant differences in cytokine levels were detected between control and patient samples for any of the inflammatory cytokines analyzed (data not shown). Viability and responsiveness of PBMC was confirmed by an increase in cytokine production in response to LPS or PMA/ionomycin (data not shown). Thus the altered levels of inflammatory mediators in sera from AFP and FP compared to controls are either produced independently of PBMC or depend on *in vivo *regulatory factors.

## Discussion

Periodic fevers are a group of disorders that belong to the recently established and growing family of autoinflammatory diseases. Periodic fevers are characterized by seemingly unprovoked, recurrent attacks of fever and severe inflammation in the absence of infectious or autoimmune etiology. The onset of disease is generally noted during childhood or, less frequently, adolescence (reviewed by [8,27-29]). Over the past two decades, advances in both the clinic and the laboratory have accelerated our understanding of autoinflammation. Within a five-year period (1997-2002), the genetic bases were discovered for each of the hereditary, monogenic periodic fevers (TRAPS, FMF, HIDS, CINCA, MWS, FCAS) that were the founding members of the autoinflammatory family. All of these diseases except TRAPS show increased secretion of IL1β due to heightened activity of the NLR family member NLRP3 (NALP3/cryopyrin; [3]). The autoinflammatory family now includes an increasing number of complex, polygenic/multifactorial diseases (including PFAPA, adult onset Still's disease, chronic recurrent multifocal osteomyelitis, Behçet's and Crohn's disease) that are of unknown etiology.

An overlap in clinical characteristics exists within the autoinflammatory family itself and to some degree with autoimmune diseases and conditions associated with recurrent infections. In this setting, the demarcation and diagnosis of specific autoinflammatory diseases can be a difficult task. The PFAPA criteria do not exclude other periodic fever syndromes [6]. Furthermore, disease entities based on criteria, like the PFAPA syndrome, might conceal different diseases from a pathophysiological perspective. In the absence of definitive markers of disease, the differential diagnosis of PFAPA syndrome can be cumbersome and sometimes uncertain. Genetic analyses for hereditary periodic fever syndrome are expensive and inaccessible in many contexts. Our experience is that genetic analyses are of limited value in patients with a typical PFAPA syndrome without additional features that suggest a hereditary periodic fever syndrome; an opinion shared by others [10]. In an attempt to reveal clues about the pathogenic mechanism(s) or biomarkers of this uncommon pediatric disease, we monitored the concentration of blood cells and serum cytokines in children with "typical PFAPA" during the asymptomatic and febrile intervals of their disease. The median age of children in this study was 4.9 years. Children in this age range are the most likely to experience 'typical PFAPA' episodes. This is an important and distinguishing feature of our study since PFAPA episodes may differ in older children that are expected to have waning disease or have 'grown out' of their episodes. We recognize that by selecting a well-defined group of patients, our cohort is relatively small thus we encourage independent confirmation of the results presented herein.

Fever in our cohort of patients was associated with an increased WBC count, due to an increase in ANC and AMC, but not ALC. We also find evidence for decreased eosinophils in the febrile period and increased thrombocytes in the afebrile interval. While the severely depreciated levels of eosinophils may be unique to PFAPA and needs further investigation, thrombocytosis may be a delayed, acute phase reaction to the previous febrile episode. It will be important to discern such differences as well as any functional changes that may correlate with alterations in cell abundance. It is also important to note that many of the observed changes in blood cell densities may be missed on a CBC analysis of blood from individual PFAPA patients due to the substantially wide range of 'normal' blood cell concentrations for children. Whether absolute cell counts that fall in the normal range but are significantly different between healthy and PFAPA children carry any biological significance remains to be proven.

The classic pro-inflammatory cytokines TNFα and IL1β are typically associated with an inflammatory response and play cardinal pathogenic roles in monogenic, hereditary periodic fevers [30]. In contrast, we did not find elevated levels of TNFα or IL1β in FP sera ~15 hours after the onset of fever. In response to *ex vivo *stimulation with LPS, PBMC from febrile patients produced more TNFα and IL1β compared to control and afebrile PBMC over a 24 hr period (data not shown). It is therefore plausible that TNFα or IL1β appeared elevated in FP sera early after the onset of fever. A similar assumption may be applicable to IFNγ since the IFNγ-inducible cytokine IP10, but not IFNγ itself, was present at elevated levels in FP sera. It is of course possible that other mechanisms for IP10 regulation are at play. We however predict that cytokines TNFα, IL1β and IFNγ rise and fall rapidly in the early hours of fever while the concentration of other cytokines (IL6 and IP10) are enhanced later in the fever period and return to control levels during the afebrile interval. The oscillations of these cytokines (TNFα, IL1β, IFNγ, IL6 and IP10) in conjunction with the diminished levels of IL4 and IL17 are indicative of a typical, IFNγ-dependent (Th1) inflammatory response (also reported by [22]).

It has been suggested that like the periodicity of inflammatory attacks in PFAPA, cytokine-producing cells in these patients may also display unique biorhythms [31]. While AMC cycled both with febrile periods and the *in vivo *expression of monokines IL6 and IP10, there was no correlation between increased AMC in FP blood and the production of cytokines by monocytes (PBMC) cultured *ex vivo*. These data suggested that PBMC in PFAPA syndrome, unlike HIDS [25] are not intrinsically activated cells and may depend on *in vivo *regulatory factors. Alternatively, cell types other than PBMC, including neutrophils, eosinophils, epithelial and endothelial cells, may be responsible for the regulation of cytokines, or other mediators, in PFAPA syndrome. Gut epithelial cells for example, are central to the pathogenesis of Crohn's disease due to dysregulated NLR signaling.

The panel of proinflammatory cytokines investigated in FP sera were present at relatively moderate levels and cycled in a manner that seemed consistent with generalized fever and a typical Th1-type inflammatory response, i.e., the cytokine profiles may not be unique to febrile episodes experienced by PFAPA patients. Future investigations require additional controls from children with other periodic fevers and acute infections. The early stages of a fever period are coincident with dynamic cytokine regulation where small variations in sampling time can yield remarkably different results. Due to technical and ethical restrictions associated with sampling at multiple time points after the onset of fever, it may be advisable to investigate cytokine profiles of disease [32] during the afebrile interval. While clinically asymptomatic, we, and others [22] demonstrate fluctuations in cytokines in the afebrile phase suggesting that the disease is active at the cellular level also between febrile flares. This is supported further by the observation of elevated levels of SAA in some patients during the afebrile period and in accordance with another study in which SAA levels fluctuated in patients with FMF that were completely asymptomatic; the authors of that studied concluded that the fluctuations were due to subclinical inflammatory activity [33]. The reduced concentrations of, for example IL7, IL17 and IFNγ that were observed in the afebrile interval may lay the foundation for future investigation into (defective) T cell regulation in PFAPA syndrome. Moreover, there is a need for investigations into the regulation and role of SAA in subclinical infection and inflammation.

## Conclusions

**Figure 5:**
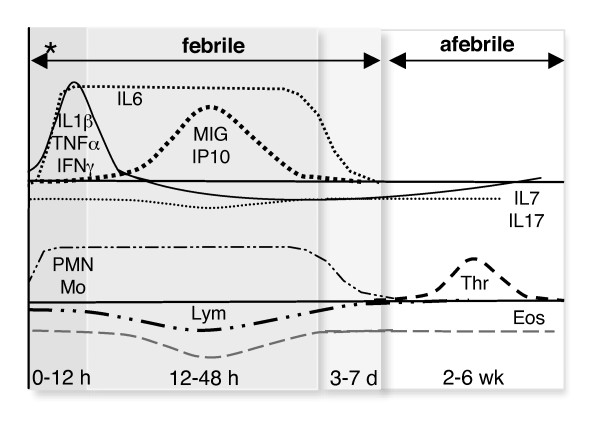
**Flux of blood cells and serum cytokines in PFAPA**. Proposed oscillations in the abundance of blood cell populations and inflammatory cytokines over time (x-axis: h, hours; d, days; wk, weeks) in a typical PFAPA cycle. Pro-inflammatory cytokines in the serum (TNFα, IL1β, IFNγ, IL6) appear early after the onset of fever [22] and are maintained (IL6) or rapidly decline (TNFα, IL1β, IFNγ) as IFNγ-induced chemokines (MIG, IP10) appear. Lymphocyte activators IL7 and IL17 may be constitutively suppressed. The febrile period is associated with increased neutrophils (PMN) and monocytes (Mo) as well as decreased lymphocytes (Lym). Eosinophils (Eos) are in sparse abundance in both febrile and afebrile intervals while thrombocytes (Thr) rise specifically in the afebrile period. * indicates the onset of fever.

Herein we report oscillations in the concentration of blood cells during the afebrile and febrile phases of typical PFAPA syndrome. An increase in circulating monocytes and thrombocytes and a major decline in circulating eosinophils are novel findings. Further, we find a relatively modest level of pro-inflammatory cytokines in the sera ~15 hours after the onset of fever with a tendency towards an IFNγ-driven inflammatory response and suppressed levels of T cell-associated cytokines during the afebrile interval. Cytokine and blood cell oscillations in PFAPA syndrome are illustrated Figure 5. Future investigations are required to conclusively discern which cells and cytokines are associated with inflammation (fever) in general and which are specific to febrile episodes in PFAPA syndrome. Advances of this nature at the cellular and molecular level will facilitate the identification of biomarkers of disease or players in disease pathogenesis.

## Abbreviations

CINCA: chronic infantile neurologic cutaneous and articular syndrome; CGD: chronic granulomatous disease; CRP: C-reactive protein; ESR: erythrocyte sedimentation rate; FCAS: familial cold autoinflammatory syndrome; FCS: fetal calf serum; FMF: familial Mediterranean fever; HIDS: hyperimmunoglobulinemia D and periodic fever syndrome; IFNγ: interferon gamma; IL1β/6: interleukin-1 beta or -6; JCA: juvenile chronic arthritis; MKD: mevalonate kinase deficiency; MWS: Muckle-Wells syndrome; NALP3: Nacht Domain-, Leucine-Rich Repeat-, and PYD-Containing Protein 3; NLR: NOD-like receptor; NLRP3: NLR family, pyrin domain-containing 3; PEST: penicillin-streptomycin (solution); RBC: red blood cell; SAA: serum amyloid A; SOJIA: systemic onset juvenile idiopatic arthritis; TNFα: tumor necrosis factor alpha; TRAPS: tumor necrosis factor receptor associated periodic syndrome; WBC: white blood cell

## Competing interests

The authors declare that they have no competing interests.

## Authors' contributions

All authors were involved in the analysis and interpretation of data and preparation of the manuscript. All authors read and approved the final manuscript. KB, PW, KS, AF, AK and SB were responsible for the conception and design of the study. Acquisition of data was done by KB, PW, VO, MS and SB. KB, PW and SB had full access to all of the data in the study and take responsibility for the integrity of the data and the accuracy of the data analysis.

## Pre-publication history

The pre-publication history for this paper can be accessed here:

http://www.biomedcentral.com/1471-2431/10/65/prepub

## Supplementary Material

Additional file 1 **Table S1: Clinical description of control children**. A table listing age, gender, allergy and reason for hospital admission of healthy children enrolled as controls in this study. Click here for file

Additional file 2 **Figure S1: Acute phase serum proteins**. The concentration of acute phase C-reactive protein (CRP, mg/L, left), serum amyloid A (SAA, mg/L, centre) and procalcitonin (μg/L, right) in sera from controls (n = 14), afebrile patients (n = 8) and patients with fever 12-18 hours (febrile, n = 6). Values are reported in Table 2. A dotted line indicates the upper and lower limits for detection for CRP and SAA or the lowest concentration of procalcitonin that may indicate an infection. Click here for file

Additional file 3 **Table S2: Complete blood count (CBC) including differential**. A table listing absolute concentrations of blood cells in healthy and PFAPA children. Click here for file

Additional file 4 **Table S3: Concentration of inflammatory mediators in serum**. The concentrations of 25 inflammatory mediators in sera from healthy children, PFAPA children in an afebrile interval or ࿄15 hours after fever commenced as determined my multiplex ELISA. Click here for file
