# Supplementary material for: Profile of blood cells and inflammatory mediators in periodic fever, aphthous stomatitis, pharyngitis and adenitis (PFAPA) syndrome
Source: BMC Pediatr. 2010 Sep 6;10:65. doi: 10.1186/1471-2431-10-65 (PMC2944328; doi:10.1186/1471-2431-10-65)
Supplement: Additional file 1 — Table S1: Clinical description of control children. A table listing age, gender, allergy and reason for hospital admission of healthy children enrolled as controls in this study. [file 1471-2431-10-65-S1.PDF]

Table S1. Clinical description of control children

ID	Age (yr; mo)	Gender	Allergy (+/-)	Reason for hospital admission
C01	1;7	M	-	Testicular surgery
C02	3;3	M	-	Inguinal hernia repair
C03	4;3	F	-	Inguinal hernia repair
C04	5;6	M	+	Inguinal hernia repair
C05	4;3	F	-	Squint surgery
C06	4;10	M	+	Inguinal hernia repair
C07	5;11	M	-	Testicular surgery & inguinal hernia repair
C08	3;10	F	-	Inguinal hernia repair
C09	6;0	M	-	Inguinal hernia repair
C10	8;1	M	-	Reconstruction of abdominal wall defect
C11	8;1	F	-	Removal of orthopaedic wire
C12	6;4	M	-	Phimosis operation
C13	3;6	M	-	Testicular surgery
C14	9;6	M	-	Removal of orthopaedic wire
