# Supplementary figures and images for: Profile of blood cells and inflammatory mediators in periodic fever, aphthous stomatitis, pharyngitis and adenitis (PFAPA) syndrome
Source: BMC Pediatr. 2010 Sep 6;10:65. doi: 10.1186/1471-2431-10-65 (PMC2944328; doi:10.1186/1471-2431-10-65)

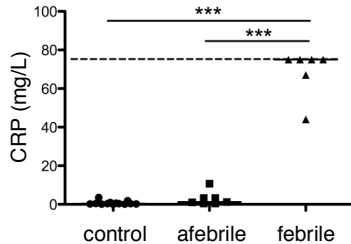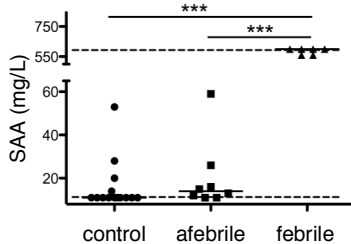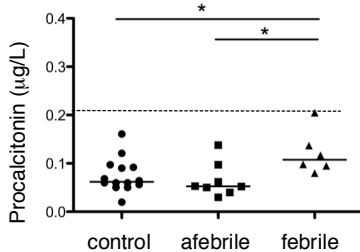

Supplement: Additional file 2 — Figure S1: Acute phase serum proteins. The concentration of acute phase C-reactive protein (CRP, mg/L, left), serum amyloid A (SAA, mg/L, centre) and procalcitonin (μg/L, right) in sera from controls (n = 14), afebrile patients (n = 8) and patients with fever 12-18 hours (febrile, n = 6). Values are reported in Table 2. A dotted line indicates the upper and lower limits for detection for CRP and SAA or the lowest concentration of procalcitonin that may indicate an infection. [file 1471-2431-10-65-S2.PDF]
