# Supplementary material for: Profile of blood cells and inflammatory mediators in periodic fever, aphthous stomatitis, pharyngitis and adenitis (PFAPA) syndrome
Source: BMC Pediatr. 2010 Sep 6;10:65. doi: 10.1186/1471-2431-10-65 (PMC2944328; doi:10.1186/1471-2431-10-65)
Supplement: Additional file 3 — Table S2: Complete blood count (CBC) including differential. A table listing absolute concentrations of blood cells in healthy and PFAPA children. [file 1471-2431-10-65-S3.PDF]

Table S2. Complete blood count (CBC) including differential

	[d]ID	[e]WBC	[f]RBC	[g]Thr	[h]Neutr	[i]Lym	[j]Mono	[k]Eos	[l]Bas
	C01	12.6	114	286	3.2	7.7	0.7	**0.92**	0.06
	C02	6.9	122	280	4.2	1.9	0.5	0.22	0.02
	C03	4.5	114	309	1.8	2.2	0.4	0.1	0
	C04	8.4	**138**	**384**	2.2	5.0	0.5	**0.68**	0.06
	C05	7.1	110	298	2.2	3.7	0.6	0.6	0
	C06	5.4	114	329	2.4	2.3	0.4	0.2	0.1
[a]control	C07	4.3	116	256	**1.4**	2.6	0.2	0.1	0
	C08	6.5	**99**	262	3.4	2.4	0.5	0.2	0
	C09	5.6	131	329	2.3	2.6	0.5	0.2	0
	C10	4.8	118	233	1.7	2.4	0.2	0.47	0.05
	C11	4.6	129	292	1.8	2.4	0.3	0.09	0.02
	C12	8.2	133	278	5.6	**1.9**	0.3	0.25	0.04
	C13	8.7	133	342	4.2	3.8	0.4	0.26	0.03
	C14	6.0	**137**	316	2.4	3.0	0.3	0.28	0.02
Mean ± SEM		6.9 ± 0.6	122 ± 3	300 ± 10	2.8 ± 0.3	3.1 ± 0.4	0.4 ± 0.0	0.32 ± 0.06	0.02 ± 0.01
	P01	7.8	115	**614**	2.1	5.2	0.4	0.07	0
	P02	6.1	117	**744**	1.9	3.7	0.4	0.07	0.02
	P03	9.1	**137**	**486**	[m]nd	nd	nd	nd	nd
[b]afebrile	P04	8.8	128	**470**	4.4	3.5	0.6	0.3	0
(AF)	P05	9.5	129	**439**	4.6	3.6	0.9	0.4	0.10
	P06	4.4	112	230	1.6	2.5	0.2	0.07	0
	P07	10.7	121	**469**	5.1	4.6	0.7	0.2	0.06
	P08	5.5	110	233	2.9	2.2	0.3	0.2	0
Mean ± SEM		7.7 ± 0.8	121 ± 3	**461 ± 61**	3.2 ± 0.5	3.6 ± 0.4	0.5 ± 0.1	0.19 ± 0.05	0.03 ± 0.01
	P05	**3.9**	133	**372**	1.7	**1.4**	0.7	**0.07**	0
	P06	5.2	110	215	3.0	**1.6**	0.6	**0.02**	0
[c]febrile	P07	11.4	117	**401**	6.8	3.5	1.0	0.05	0.05
(F)	P08	14.4	112	257	**11.8**	2.0	0.6	**0.02**	0
	P09	12.2	129	319	**9.2**	2.1	0.7	**0.04**	0.10
	P10	12.2	125	nd	**10.0**	**1.4**	0.8	**0.01**	0.10
Mean ± SEM		9.9±1.7	121 ± 4	313 ± 35	7.1 ± 1.6	2.0 ± 0.3	0.7 ± 0.1	**0.04 ± 0.0**	0.04 ± 0.02
	P03	7.9	133	**371**	4.7	2.2	0.7	0.2	0.1
	P04	12.7	119	226	**8.8**	2.2	**1.5**	**0**	0

Data shown as scatter plots in Figure 1 and Figure 2
[a-d]Concentration of blood cells in [a]healthy children and PFAPA children in either an [b]afebrile interval or [c]within the first 24 hours of a febrile episode. Samples from FP03 and FP04 were drawn respectively ~ 12 hours before and ~120 hours after fever appeared and were excluded from the calculation of mean cell concentration ± SEM. Numerical digits in the assigned [d]identification number (ID) are unique to individuals. Values in bold are outside the range for healthy children (www.kliniskkemi.se).
[e-l] Expected cell counts x 10^9/L: [e]White blood cells (WBC) 4.5-15, [g]Thrombocytes [33] 150-350, [h]Neutrophils (Neutr)1.5-8.5, [i]Lymphocytes (Lym) 2-9.5, [j]Monocytes (Mono) 0.1-1, [k]Eosinophils (Eos) 0.04-0.4, [l]Basophils (Bas) 0-0.1, [f]Red blood cells (RBC) as Hemoglobin (Hb) g/L 105-135.
[m]nd, not determined.
