# Supplementary material for: Profile of blood cells and inflammatory mediators in periodic fever, aphthous stomatitis, pharyngitis and adenitis (PFAPA) syndrome
Source: BMC Pediatr. 2010 Sep 6;10:65. doi: 10.1186/1471-2431-10-65 (PMC2944328; doi:10.1186/1471-2431-10-65)
Supplement: Additional file 4 — Table S3: Concentration of inflammatory mediators in serum. The concentrations of 25 inflammatory mediators in sera from healthy children, PFAPA children in an afebrile interval or ࿄15 hours after fever commenced as determined my multiplex ELISA. [file 1471-2431-10-65-S4.PDF]

Table S3. Concentration of inflammatory mediators in serum

Analyte	[e]Range (pg/ml)	[f,g]SD	[a]control			[b]afebrile (AF)			[c]febrile (F)		
			[d]C04	C05	C08	P03	P04	P05	P05	P09	P10
Classic pro-inflammatory											
IL1β	18 - 12810		45	*12*	47	32	*24*	[h]<	<	<	*14*
IL1Ra	42 - 30900		1085	683	941	758	372	452	1587	702	1806
TNFα	8 - 5600	*	23	19	23	17	19	18	*14*	18	18
IL6	10 - 6900	**	*11*	*7*	23	*9*	*6*	*17*	64	35	44
Growth stimulators											
GMCSF	28 - 20590		61	*18*	*27*	<	<	72	167	<	49
IL2	9 - 6430		30	*12*	21	19	*13*	*8*	*13*	*12*	25
IL2R	34 - 24660		1159	1159	1257	810	1126	2030	1451	1233	1216
1L7	18 - 13070	**	67	52	62	*32*	*34*	*32*	*19*	43	*29*
T_H1-associated											
IFNγ	17 - 12700	*	143	103	125	96	91	94	80	99	88
IFNα	10 - 7750		116	92	116	83	89	86	86	89	125
IL12p40p70	14 - 10500		1409	1564	1505	1367	1402	1118	1052	1393	1434
T_H2-associated											
IL4	10 - 7580		222	214	213	182	227	191	194	204	192
IL5	33 - 24170		*11*	*7*	*12*	*7*	*7*	*6*	*5*	*7*	*7*
IL10	19 - 13910		*8*	*4*	*6*	*5*	*3*	*10*	*35*	*5*	*6*
IL13	15 - 10850	*	43	*18*	47	*12*	<	<	<	*1*	<
IL15	26 - 18670		49	*17*	*33*	*25*	*6*	*5*	*14*	*8*	75
T_H17											
IL17	33 - 23720	*	199	177	206	166	143	155	147	162	166
Chemotactic											
CCL2/MCP1	17 - 12180		715	746	708	1055	606	754	979	507	777
CCL3/MIP1α	12 - 8440		128	105	118	97	112	101	125	103	127
CCL4/MIP1β	16 - 11830	*	176	138	145	124	129	136	201	174	162
CCL5/RANTES	11 - 8380		>	[i]>	>	>	>	>	*23558*	>	*81711*
CCL11/Eotaxin	6 - 4430	*	226	156	136	210	193	190	141	106	49
CXCL8/IL8	7 - 5080		67	12	17	*7*	22	14	38	20	<
CXCL9/MIG	4 - 2890		113	91	105	108	77	110	1078	94	256
CXCL10/IP10	3 - 2540		40	87	64	53	63	110	633	107	243

Select data shown in Figure 3 and Figure 4

[a-d]Concentration of inflammatory mediators in sera from [a]healthy children (control), PFAPA children in an [b]afebrile interval (AF) or [c] ~15 hours after fever commenced (F). Numerical digits in the assigned [d]identification number (ID) are unique to individuals.

[e]Lower and upper limits of detection (pg/ml) for each analyte as reported by the manufacturer.

[f]SD, a statistical difference in analyte concentration between the groups was determined by one-way ANOVA with a p-value < 0.05 (*), < 0.01 (**) and < 0.001 (***).

[g]Values in italics were extrapolated beyond the standard range (StarStation software) and excluded from statistical analysis.

[h,i]< and > indicate a concentration of the analyte below or above the detectable range.
